# *Microbacterium paraoxydans* Bloodstream Infection in a Patient with Long-Term Indwelling Central Venous Catheter: A Case Report and Literature Review

**DOI:** 10.3390/microorganisms14092040

**Published:** 2026-09-12

**Authors:** Yi-Kai Huang, Po-Hsiu Huang, Chien-Hao Tseng, Chia-Wei Liu, Wei-Hsuan Huang, Hsien-Po Huang, Po-Yu Liu, Ting-Kuang Yeh

**Affiliations:** 1 Department of Internal Medicine, Taichung Veterans General Hospital, Taichung 407, Taiwan; 2 Division of Infectious Diseases, Department of Internal Medicine, Taichung Veterans General Hospital, Taichung 407, Taiwan; 3 School of Medicine, College of Medicine, National Yang Ming Chiao Tung University, Taipei 112, Taiwan; 4 Institute of Molecular Biology, National Chung Hsing University, Taichung 402, Taiwan; 5 Graduate Institute of Biomedical Engineering, National Chung Hsing University, Taichung 402, Taiwan; 6 Institute of Clinical Medicine, National Yang Ming Chiao Tung University, Taipei 112, Taiwan; 7 Infection Control Center, Taichung Veterans General Hospital, Taichung 407, Taiwan; 8 Department of Post-Baccalaureate Medicine, College of Medicine, National Chung Hsing University, Taichung 402, Taiwan; 9 Doctoral Program in Translational Medicine, Rong Hsing Translational Medicine Research Center, National Chung Hsing University, Taichung 402, Taiwan

**Keywords:** *Microbacterium paraoxydans*, *Microbacterium*, bloodstream infection, immunocompromised status

## Abstract

*Microbacterium* species, a genus of aerobic Gram-positive coryneform rods, are rare causes of human infection with only a few sporadic cases reported worldwide. MALDI-TOF MS, 16S rRNA gene sequencing, and even whole-genome sequencing are used to identify the species in a clinical laboratory setting. The study reports a case of *Microbacterium paraoxydans* bloodstream infection and reviews the literature, including eight additional reported cases of *M. paraoxydans* infection. We summarize the characteristics of each case, highlight the identification methods, and identify recurrent characteristics for *M. paraoxydans* infection. A 65-year-old man presented with stable condition of esophageal cancer and long-term prednisolone therapy for psoriasis. A central venous catheter (CVC) was placed for years for treatment of esophageal cancer. The patient presented with fever and chills, and *Microbacterium paraoxydans* was identified by MALDI-TOF MS from blood culture samples via chemo port. After the chemo port was removed, vancomycin followed by levofloxacin was administered for the entire 14-day treatment course according to susceptibility tests. This study highlights that a long-term central venous catheter and immunocompromised status were common characteristics among the reported cases of *Microbacterium* spp. infection. The susceptibility of *Microbacterium* spp. is not fully characterized and varies across reports; gentamicin and trimethoprim/sulfamethoxazole appeared more consistently active, whereas susceptibility to vancomycin was variable. We report this case to highlight the increasing number of infections caused by *Microbacterium* spp. in the current era and to provide information on the treatment of *M. paraoxydans*.

## 1. Introduction

*Microbacterium* is a genus of aerobic Gram-positive coryneform rods usually found in the environment, such as soil, wastewater, and animals [1]. *Microbacterium* species were usually identified based on their morphological and biochemical profiles, but the discriminating power sometimes had limitations, which made the identification more challenging. Since the 2010s, matrix-assisted laser desorption ionization time-of-flight mass spectrometry (MALDI-TOF MS) has been widely used in health-care settings due to rapid sample detection and colony identification, despite the fact that there are some drawbacks, such as misidentifications between similar species [2]. Later, 16S rRNA gene sequencing became a useful tool to identify different *Microbacterium* species. In recent years, with the development of next-generation sequencing techniques, whole-genome sequencing (WGS) could be used to confirm the clinical *Microbacterium* spp. isolate [3]. To date, hundreds of species in the *Microbacterium* genus have been isolated, but as for clinical settings, these species are rarely human pathogens [4,5]. Only a few cases were reported that led to human infection, and most were associated with catheter-related bloodstream infection and peritoneal dialysis-related peritonitis [6,7,8]. However, an increasing number of infections have recently been identified and reported worldwide, particularly in immunocompromised hosts. Here we report a case of bloodstream infection of *Microbacterium paraoxydans* from a 65-year-old male with past medical history of psoriasis and esophageal cancer in stable status, who had an indwelling central venous catheter.

## 2. Case Report

The patient was a case of esophageal cancer receiving concurrent chemoradiotherapy (CCRT) with now stable status and psoriasis undergoing long-term corticosteroid therapy (prednisolone 5 mg/day). He had a long-term indwelling chemo port for 4 years. He was admitted for fever survey due to nausea and vomiting with recurrent fever up to 38.3 °C for one week. Laboratory tests reported WBC 9030/µL with 83.4% of neutrophils and markedly elevated CRP (22.96 mg/dL). On admission, three blood culture sets were collected—two from the chemo port and one from a peripheral vein. Both chemo-port sets grew Gram-positive bacilli identified as *M. paraoxydans* by VITEK^®^ MS PRIME (bioMérieux, Durham, NC, USA), whereas the peripheral set showed no growth. Review of his records revealed that, during a febrile episode a few months earlier, two peripheral blood culture sets had been obtained and one had grown *M. paraoxydans* with an identical antimicrobial susceptibility profile; he was discharged against medical advice before adequate treatment and was lost to follow-up. During this hospitalization, abdominal sonogram and chest computed tomography revealed no obvious infection source. The echocardiogram did not detect significant abnormality or vegetations. The susceptibility test was determined by using Mueller–Hinton agar (CMP, Taiwan) for disk diffusion and interpreted using the Clinical and Laboratory Standards Institute (CLSI) guidelines, revealing that the *M. paraoxydans* isolate was resistant to penicillin, but susceptible to ciprofloxacin, vancomycin, clindamycin, gentamicin, and trimethoprim/sulfamethoxazole. Under the diagnosis of a bloodstream infection with a suspected catheter source (*M. paraoxydans* bacteremia), we therefore removed the chemo port and completed antibiotic treatment starting with vancomycin, followed by levofloxacin for a total of two weeks. The subsequent blood culture did not yield the growth of *M. paraoxydans*. The patient was discharged in stable condition.

## 3. Discussion

We present an unusual case of suspected catheter-related bloodstream infection due to *M. paraoxydans* in a patient with oncologic and autoimmune comorbidities. *M. paraoxydans* has been recovered from various environmental sources, such as soil and water. Bacteremia due to *M.* paraoxydans has rarely been reported worldwide.

A literature search was conducted in PubMed using the terms “Microbacterium species,” “Microbacterium paraoxydans,” and “human infection” to identify previously reported human *M. paraoxydans* infections; eight cases with available clinical courses were retrieved (Table 1). Regarding the clinical characteristics, among the reported bloodstream infection cases, long-term central venous catheterization and immunocompromised status were frequently observed. Two cases were reported as hematologic disease, and the others were diagnosed as sarcoidosis and congenital intestinal enteropathy [1,3,4,5]. As for other possible risk factors, according to an outbreak investigation published in 2001, having a previous bacteremia with a different organism in the preceding 14 days was associated with an increased risk of *Microbacterium* species bacteremia among oncology patients with a central venous catheter [9].

Regarding the identification methods of *Microbacterium* species, as listed in Table 1, most previous cases used 16S rRNA gene sequencing and confirmed its utility for identifying *Microbacterium paraoxydans*. Only one case previously reported performing matrix-assisted laser desorption ionization time-of-flight mass spectrometry (MALDI-TOF MS) followed by whole-genome sequencing (MiSeq Genetic Analyzer, Illumina) to confirm the clinical isolate [3]. Some reports noted that MALDI-TOF MS was occasionally unable to provide species-level identification [5]. Infection by *Microbacterium* spp. may be underestimated because detailed identification of Gram-positive bacilli is not always performed when the conventional biochemical method does not confirm the organism. In our present case, we used MALDI-TOF MS to confirm the clinical isolate.

The antimicrobial susceptibility test (AST) results are listed in Table 2. Among the reported cases, antimicrobial resistance varied. Only one case was reported to be susceptible to penicillin, and the rest were reported intermediate or resistant. As for treatment, glycopeptides, such as vancomycin and teicoplanin, are often used for the treatment of *Microbacterium* spp. bacteremia. However, in the case of *M. paraoxydans*, data are limited owing to the small number of reports. Among our review of the literature, vancomycin or teicoplanin was reported to be susceptible in two cases and administered in four with successful treatment. Returning to our case, the susceptibility test demonstrated that the *M. paraoxydans* isolate was resistant to penicillin but susceptible to ciprofloxacin, vancomycin, clindamycin, gentamicin, and trimethoprim/sulfamethoxazole. Vancomycin followed by levofloxacin was administered for 14 days in our case, and the following blood cultures did not yield further bacterial growth. In all reported catheter-related bloodstream infection cases, the catheter was removed. Among the peritoneal dialysis-related cases, two were cured with antimicrobial therapy alone, whereas one required catheter removal after recurrence. In our case, we also removed the chemo port.

In addition to bloodstream or catheter-related infection, *M. paraoxydans* was also reported in three cases, which were all ESRD patients undergoing long-term peritoneal dialysis [6,7,8]. Two of the peritoneal dialysate cultures yielded *Microbacterium* species, and 16S rRNA gene nucleotide sequencing identified the organism as *M. paraoxydans*, and the other utilized a MALDI-TOF MS system for identification. Peritoneal dialysis-related peritonitis due to *M. paraoxydans* was diagnosed. Based on the results of the antibiotic susceptibility test, one was treated with intraperitoneal vancomycin and oral clarithromycin, one received intravenous administration of erythromycin and oral administration of sulfamethoxazole/trimethoprim, and intraperitoneal cefazolin and ceftazidime were administered in the other case for a 2-week treatment course. The outcome of *M. paraoxydans* infection was favorable when the focus of the infection was CRBSI or peritonitis. No fatal cases were reported.

In this case report, we did have some limitations. First, time to positivity and differential time to positivity between catheter and peripheral samples were not measured. Neither the criteria for a definitive diagnosis of CRBSI nor those for a possible CRBSI as defined by the IDSA (the latter requiring paired quantitative cultures from two catheter lumens) could be fulfilled, because quantitative blood cultures, differential time to positivity, and a catheter-tip culture were not analyzed [11]. The episode was nonetheless a clinically suspected catheter-related bloodstream infection and was consistent with the CDC/NHSN surveillance definition of a central line-associated bloodstream infection [12]. Second, 16S rRNA gene sequencing was not conducted for confirmatory molecular identification; it could not be performed in the current condition due to lack of isolate. Third, no *Microbacterium*-specific susceptibility breakpoints have been established; interpretation followed the CLSI M45 criteria for *Corynebacterium* spp. and related coryneform Gram-positive rods, which M45 in turn derives from the corresponding CLSI M100 breakpoints, and the categorical results should therefore be interpreted with some caution [13].

In conclusion, *Microbacterium* species are mostly detected in the environment and rarely lead to human infection. A long-term central venous catheter was a common feature among the reported cases of *Microbacterium* spp. infection. Among *Microbacterium* spp. infection, *M. paraoxydans* is a rare cause of human infection, and data to guide the choice and efficacy of antimicrobial therapy remain scarce. The patient in our case was treated with intravenous vancomycin followed by levofloxacin based on the antimicrobial susceptibility test and cured after a 14-day course.

## Figures and Tables

**Table 1:** Summary of cases with *Microbacterium paraoxydans* infection.

Case	Age	Gender	Underlying Diseases	Focus of the Infection	Indwelling Catheter	Remove Catheter	Final Identification	Treatment	Outcome
1 [1]	5	M	ALL	CRBSI	Y	Y	16S rRNA gene sequencing	IV AMP	Cured
2 [4]	15	F	Congenital gut enteropathy	CRBSI	Y	Y	16S rRNA gene sequencing	IV TEC 10d	Cured
3 [6]	60	M	Graves disease, ESRD under CAPD	Peritonitis	Y	N	16S rRNA gene sequencing	IV ERY 14d + PO TMP/SMX 21d	Cured
4 [7]	54	F	DM, ESRD under CAPD	Peritonitis	Y	N	16S rRNA gene sequencing	Intraperitoneal VAN + PO CLA	Cured
5 [3]	61	F	Sarcoidosis, CHF, HTN	CRBSI	Y	Y	MALDI-TOF MS, WGS	PO LEV+ IV VAN	Cured
6 [5]	11	F	Acute leukemia, neutropenia	CRBSI	Y	Y	16S rRNA gene sequencing	IV TZP + TEC	Cured
7 [8]	74	M	ESRD under CAPD, cirrhosis, DM, undiagnosed pancreatic mass	Peritonitis	Y	Y	MALDI-TOF MS	IP CFZ + CAZ 14d	Cured
8 [10]	late 60s	F	Endometrial carcinoma	Endocarditis	Y	Y	MALDI-TOF MS	AMP/SBT + CRO	Cured
9 (this case)	65	M	Esophageal cancer, psoriasis	Suspected CRBSI	Y	Y	MALDI-TOF MS	IV VAN + LEV	Cured

CRBSI: catheter-related bloodstream infection, CFZ: cefazolin, AMP: ampicillin, VAN: vancomycin, LEV: levofloxacin, CRO: ceftriaxone, ERY: erythromycin, CAZ: ceftazidime, TEC: teicoplanin, TZP: piperacillin-tazobactam, CLA: clarithromycin, TMP/SMX: trimethoprim/sulfamethoxazole. Focus-of-infection classifications for Cases 1–8 are those reported by the original authors; for the present case (Case 9), the episode was a suspected CRBSI (see Limitations). For Cases 3, 4 and 7 the indwelling catheter was a peritoneal dialysis catheter; in the remaining cases it was a central venous catheter.

**Table 2:** Antimicrobial susceptibility testing (AST) of *Microbacterium paraoxydans* isolates.

	Our Case	1 [1]	2 [4]	3 [6]	4 [7]	5 [3]	6 [5]	7 [8]
Penicillin	R	I (1.5–2)	R		S (1.0)	I (0.75)	I (1)	R (4)
AMP		(1–1.5)				(0.5)		
VAN	S	NS (3–4)	S (3)		S (2.0)	NS (3)	R (≥2)	NS (4)
TEC			S (3)					
TMP/SMX	S		S (0.125)	S				S (≤0.5)
GEN	S	S (2–3)	S (1)		S (3.0)	S (2)		S (≤2)
CIP	S	I (1–1.5)	S (1)			I (1.5)		I (2)
ERY				S	S (0.064)	R (32)	R (≥4)	S (≤0.25)
CLI	S		R			R (4)		
AST methods	Disk diffusion	E-test	Disk diffusion, E-test	E-test	E-test	BD Phoenix system	Broth microdilution, E-test	Serial microbroth dilution

Data are presented as the interpretive category, followed where reported by the minimum inhibitory concentration (MIC) in µg/mL in parentheses (e.g., R (4) = resistant, MIC 4 µg/mL; S (≤0.5) = susceptible, MIC ≤ 0.5 µg/mL); a hyphenated value indicates the MIC range across isolates. Because no *Microbacterium*-specific breakpoints exist, interpretive categories follow the criteria reported in each cited study or, where none were reported, CLSI M45 (coryneform Gram-positive bacilli). AST methods differ among studies (see the “AST methods” row); results obtained by heterogeneous methods are therefore not directly comparable. AMP: ampicillin; GEN: gentamicin; VAN: vancomycin; CIP: ciprofloxacin; ERY: erythromycin; TEC: teicoplanin; TMP/SMX: trimethoprim/sulfamethoxazole; CLI: clindamycin; S: susceptible; R: resistant; NS: non-susceptible; I: intermediate. Blank entries: not tested. Interpretive criteria differed across studies. Case 1 [1] reported MIC ranges for six strains (the case isolate plus five others) without categories; these were assigned by the present authors using CLSI M45, 3rd ed. Case 2 [4] used CLSI M45-A (2006), under which vancomycin at 3 µg/mL is susceptible (≤4) but would be non-susceptible by the 3rd ed. (≤2). Case 4 [7] reported penicillin at 1.0 µg/mL as susceptible, which the 3rd ed. classifies as intermediate.

## Data Availability

The original contributions presented in this study are included in the article. Further inquiries can be directed to the corresponding author.
